# A New Spiking Convolutional Recurrent Neural Network (SCRNN) With Applications to Event-Based Hand Gesture Recognition

**DOI:** 10.3389/fnins.2020.590164

**Published:** 2020-11-17

**Authors:** Yannan Xing, Gaetano Di Caterina, John Soraghan

**Affiliations:** Neuromorphic Sensor Signal Processing Laboratory, Centre for Signal and Image Processing (CeSIP), Department of Electronic and Electrical Engineering, University of Strathclyde, Glasgow, United Kingdom

**Keywords:** spiking neural network, DVS, gesture recognition, event-based processing, video processing

## Abstract

The combination of neuromorphic visual sensors and spiking neural network offers a high efficient bio-inspired solution to real-world applications. However, processing event- based sequences remains challenging because of the nature of their asynchronism and sparsity behavior. In this paper, a novel spiking convolutional recurrent neural network (SCRNN) architecture that takes advantage of both convolution operation and recurrent connectivity to maintain the spatial and temporal relations from event-based sequence data are presented. The use of recurrent architecture enables the network to have a sampling window with an arbitrary length, allowing the network to exploit temporal correlations between event collections. Rather than standard ANN to SNN conversion techniques, the network utilizes a supervised Spike Layer Error Reassignment (SLAYER) training mechanism that allows the network to adapt to neuromorphic (event-based) data directly. The network structure is validated on the DVS gesture dataset and achieves a 10 class gesture recognition accuracy of 96.59% and an 11 class gesture recognition accuracy of 90.28%.

## 1. Introduction

During the past couple of decades, computer vision applications have become increasingly important in many industrial domains such as security systems, robotics, and medical devices. Many Deep Neural Network (DNN) based algorithms have outperformed human performance in different image recognition tasks such as the success of Convolutional Neural Networks (CNN) (Krizhevsky et al., 2012) in the 2012 ILSVRC image classification challenge. However, it remains a challenge to extend the achievements in static image recognition to dynamic scene recognition, which has both strong temporal and spatial correlations. Human hand gesture recognition is one such problem that is significant for human-computer interaction (Mitra and Acharya, 2007; Rautaray and Agrawal, 2012; Haria et al., 2017). The hand's movement conveys certain information that can be used as a tool to communicate with computers. The hand gesture recognition has shown a significant value in applications such as virtual reality (Wickeroth et al., 2009; Frati and Prattichizzo, 2011), robot control (Droeschel et al., 2011; Liu and Wang, 2018), and sign language recognition (Liang and Ouhyoung, 1998; Yang et al., 2010; Pigou et al., 2015). The importance of developing intelligent models for complex Spatio-temporal processing is widely recognized for solving dynamic scene based recognition problems. In recent years, recurrent neural network (RNN) structures such as the long-short-term-memory (LSTM) (Hochreiter and Schmidhuber, 1997) have been shown to be effective for time-based sequence to sequence classification and prediction tasks. However, the LSTM is still inherently inefficient for the dynamic scene recognition since it does not deal with any spatial information. Research has shown the effectiveness of combining the recurrent structure and convolution operation in the dynamic scene recognition such as CNN-LSTM structure (Donahue et al., 2017; Wang et al., 2017) and convLSTM structure (Shi et al., 2015; Song et al., 2018; Zhou et al., 2018). Such a mechanism allows feature extraction to use both temporal and spatial information.

Concerning the data acquisition side, the traditional vision sensor is a digital camera that repeatedly refreshes its entire array of pixel values at a predefined frame rate. However, using the digital camera has three drawbacks for dynamic motion recognition. First, a digital camera normally operates with a predefined frame sampling rate (typically range 25–50 frames per second), which limits the temporal resolution of activities observed. Second, consecutive frames and redundant pixels in each frame waste significant storage resources and computation. Third, the dynamic range of traditional image sensors is limited by its exposure time and integration capacity. Most cameras suffer from saturating linear response with dynamic range limited to 60–70 dB where light from natural scenes can reach approximately 140 dB of the dynamic range (Posch et al., 2011). The dynamic vision sensor (DVS) (Lichtsteiner et al., 2008; Posch et al., 2011; Brandli et al., 2014) provides a solution to these problems. The DVS using address event representation (AER) is an event-driven technology based on the human visual system. The benefit of the event-based sensor on the dynamic scene recognition task is that it offers very high temporal resolution when a large fraction of the scene changes, which can only be matched by a high-speed digital camera with the requirement of high power and significant resources.

In DVS, information is coded and transmitted as electric pulses (or spikes), which is similar to the processing mechanism in biological sensory systems. The output of DVS is generated asynchronously by comparing each activity of a retina pixel with a certain threshold. The emergence of dynamic vision sensor (DVS) (Lichtsteiner et al., 2008) demonstrated significant potential in applications of ultra-fast power efficient computing. Compared to traditional vision sensors, DVS returns unsynchronized events rather than a sampled time-based frame series. For a given real-world input, DVS records only changes in pixel intensity values and outputs a stream of ON/OFF discrete events regarding the changing polarity. Such an event-based acquisition mechanism offers many advantages such as low power consumption, less redundant information, low latency, and a high dynamic range. Despite the advantages of DVS, it is still challenging to apply the traditional computer vision algorithms to unsynchronized DVS output data.

The spiking neural network (SNN) provides an efficient solution to event-based data processing. As the DVS mimics the biological retina, the spiking neural network (SNN) mimics the human brain's functionality by utilizing bio-inspired neuron and synapse models. The major difference between SNN and traditional ANNs is the information carrier between their fundamental processing units. The SNN propagates only individual spikes rather than floating-point numbers. Such a characteristic provides an effective and low power computing strategy for event-driven inputs. Previous work has demonstrated application examples of combining SNN and the event-based visual sensor such as extracting car trajectories on a freeway [10], recognition of human postures (Pérez-Carrasco et al., 2010; Jiang et al., 2019), object tracking (Hinz et al., 2017), and human gesture recognition (Amir et al., 2017). However, to our knowledge and to date, the convolutional recurrent network structure which has been particularly designed for gesture recognition has not been widely investigated in the SNN domain. Wang W. et al. (2019) presented a spiking recurrent neural network used for action recognition, but the term “spiking” in their work does not represent the event-based processing but a spiking signal that was used to help a traditional RNN correct its contaminated memory. Demin and Nekhaev (2018) proposed a bio-inspired learning rule FEELING with an attempt on the recurrent structure, which is applied to the handwritten digit recognition. The FEELING algorithm was further implemented by Nekhaev and Demin (2020) with an convolutional recurrent structure that has been proven to be more energy efficient on hand digit recognition. However, this work did not consider the research line where the combination of convolutional and recurrent structure is more significant in a dynamic scene based recognition (i.e., hand gesture recognition). Furthermore, this work ignored the adaptability of SNN with neuromorphic hardware and sensors.

In this paper, we present a novel spiking neural network structure that can adapt to the neuromorphic vision data-based recognition problem especially for data that contains strong spatiotemporal correlations such as human hand gesture recognition. The convolutional operation and recurrent neural network connections are combined in an SNN that uses a supervised learning based spiking convolutional recurrent neural network (SCRNN). By adjusting the integration period of the input data sequence and convolution kernel, SCRNN can achieve arbitrary Spatio-temporal resolution related to the recognition demand. Moreover, The Spike Layer Error Reassignment (SLAYER) training algorithm (Shrestha and Orchard, 2018) is successfully deployed to the SCRNN for the purpose of generalization and training stability. It utilizes both temporal error and axonal delay credit assignment to minimize the computational complexity. The use of SLAYER effectively prevents the common gradient vanishing and explosion problem associated with recurrent neural networks. Since the recurrent propagation between the SCRNN cells relies on the information fusion from inputs of current timestamps and output from previous timestamps, particularly for SCRNN, a spiking feature map integration method is developed in the SCRNN cell to maintain information continuity in the temporal domain. Furthermore, The SCRNN is validated by a series of experiments on the DVS gesture dataset (Amir et al., 2017) to prove its robustness for the motion-based neuromorphic action recognition problem.

The remainder of this paper is organized as follows. Section 2 introduces the related work in the spiking recurrent neural network and SLAYER training algorithm. In section 3, detailed descriptions are provided in terms of individual SCRNN cell and overall SCRNN topology. The experiment results on the DVS gesture dataset is presented and discussed in section 4. The experiment result is analyzed and compared with previous work. Finally, the conclusions are provided in section 5.

## 2. Preliminaries

This section provides an explanation of the background of SNN, the SLAYER training algorithms (Shrestha and Orchard, 2018), as well as relevant previous works on convolutional recurrent neural networks.

### 2.1. Spiking Neural Network

In recent years, deep learning technologies have rapidly revolutionized the field of machine learning. Traditional deep neural networks are trained using supervised learning algorithms, which are usually based on gradient descent backpropagation. A neural network comprises several fundamental computing units (neurons) containing a weighted and biased continuous activation function. Typical examples of these activation functions include sigmoid, hyperbolic tangent and ReLU (Nair and Hinton, 2010). With the feed-forward and recurrent structure, this computation strategy allows them to be able to approximate any analog function universally (Vreeken, 2002).

Although DNNs were initially brain-inspired, their structure, neural information processing and learning method are still fundamentally different from the brain. One of the most distinctive differences is the means in which information is carried between neurons. That is one of the main reasons for the increased interest in spiking neural networks (SNNs). SNN raises the level of biological realism of ANNs by utilizing individual spikes as information carriers. This allows the network computation and communication to incorporate spatial-temporal information. The spikes used in SNN, however, are sparse in time with uniform amplitude, but rich in their information content when they occur in time. The information in SNNs is presented by spike timing e.g., latency, frequency or the population of the neuron that are emitted spikes (Gerstner et al., 2014).

**Figure 1:**
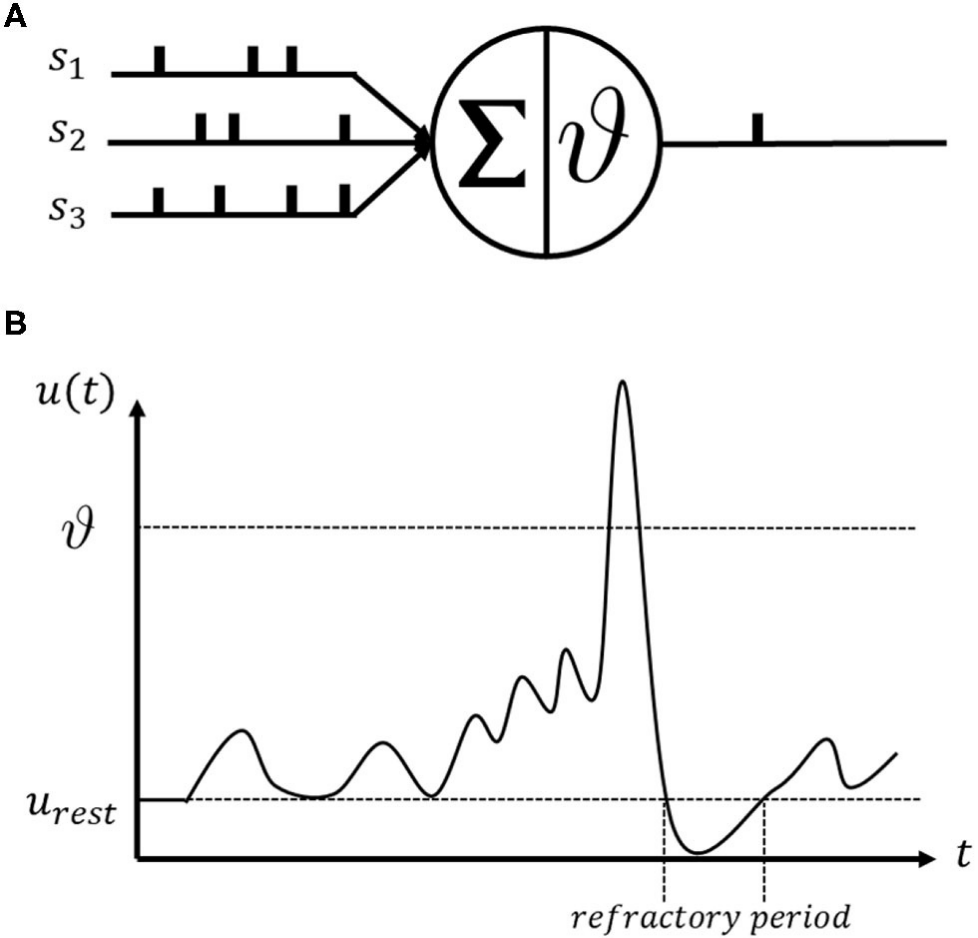
The illustration of the spiking neuron operating mechanism. **(A)** An example of a single spiking neuron that consists of integrator and threshold operator. **(B)** A simulation of membrane potential *u*(*t*) change of a spiking neuron.

The SNN is an ideal universal spike generation model that mimics the actual biophysical mechanisms described by Hodgkin and Huxley (Hodgkin and Huxley, 1990). The spikes are only identified at the time instant when they arrive at the post-synaptic neuron. Non-linear differential equations are commonly used in SNN neuron modeling to generate the membrane potential through time (Hodgkin and Huxley, 1990; Abbott, 1999; Gerstner, 2009; Teka et al., 2014). Figure 1 illustrates the basic operating mechanism of a spiking neuron. This illustrates a single spiking neuron that receives incoming spike trains from *s*_1_, *s*_2_, and *s*_3_ and generates an output spike as shown in Figure 1A. The incoming spikes to a neuron are integrated and transferred to the membrane potential dynamics *u*(*t*) as shown in Figure 1B. Whenever the membrane potential reaches a certain threshold value ϑ, the spiking neuron will emit a spike and reset the membrane potential to its resting value *u*_*rest*_. After a spike activity, the neuron enters the refractory period and cannot fire any further spikes until its membrane potential resets to its resting value.

A typical spiking neuron model can contain additional parameters that approximate the membrane potential dynamics in the neural cortex. Spiking neuron models commonly used in SNNs include: Integrate and fire neurons (IF) (Feng and Brown, 2000; Feng, 2001), Leaky integrated and fire neurons (LIF) (Liu and Wang, 2001), Hodgkin-Huxley model (Bower et al., 1995) and Spike Response Model (SRM) (Gerstner, 2008) etc.

Recent research has successfully demonstrated examples of SNN based applications including object recognition (Diehl and Cook, 2015; Kheradpisheh et al., 2018), speech processing (Stéphane et al., 2005; Wysoski et al., 2010; Tavanaei and Maida, 2017), pattern recognition (Han and Taha, 2010; Dhoble et al., 2012; Mohemmed et al., 2012; Kasabov et al., 2013). Furthermore, many developed neuromorphic computing platforms have demonstrated tremendous potential in real-world power limited applications. The IBM TrueNorth systems consist of 5.4 billion transistors with only 70mW power density consumption, which accounts for only 1/10,000 of traditional computing units (Akopyan et al., 2015). The SpiNNaker platform (Furber et al., 2013, 2014) developed by Researchers in Manchester provides ASIC solutions to hardware implementations of SNNs. It utilizes multiple ARM cores and FPGAs to configure the hardware and PyNN (Davison et al., 2009) software API to enable the scalability of the platform. The Loihi NM chip (Davies et al., 2018) is a digital NM computing platform that was recently announced by Intel. One of the most attractive features of Loihi is the potential of online-learning. Loihi has a special programmable microcode engine for SNN training on the fly. The emergence of these hardware technologies demonstrates strong suitability of applying power efficient neuromorphic computing into real-world mobile units.

### 2.2. Spike Layer Error Reassignment in Time (SLAYER)

Currently, the training procedure of most ANNs relies on the combination of continuously differentiable activation function and a gradient descent convergence algorithm. Spiking Neural Networks are similar to traditional neural networks in topology but differ in the way of information carrier and the choice of neuron models. The non-differentiable nature of biological-plausible spiking neurons is the main challenge of the development of SNN training algorithms. Spike Layer Error Reassignment in Time (SLAYER) alternatively approximates the derivative of the spike function based on the neuron state changes and assigns the error to previous layers. A description of the SLAYER training algorithm is provided in the next subsection.

The neuron model used for the SLAYER is the Spike Response Model (SRM). The membrane potential generation process of a SRM neuron is achieved by convolving a spike response kernel σ(*t*) with the incoming spike train *s*_*i*_(*t*) to this neuron to form a spike response signal as *a*(*t*) = (σ(*t*)*s_*i*_(*t*)). Here the index *i* represents the *i*_*th*_ input channel. The spike response signal is further weighted by the synaptic weight *w*. Similarly, the refractory response signal can be obtained *via* convolving a refractory kernel ν(*t*) with the neuron output spike train *s*_*o*_(*t*) as *r*(*t*) = (ν(*t*)*s_*o*_(*t*)). The overall neuron membrane potential *u*(*t*) can be obtained by summing all the spike response signal and refractory response signal as:

(1) $$\begin{array}{r} \begin{array}{c} u\left(t\right)=\sum w_{i}\left(\sigma \left(t\right)*s_{i}\left(t\right)\right)+\left(\nu \left(t\right)*s_{o}\left(t\right)\right)\\ \;={\text{W}}^{⊤}\text{a}\left(t\right)+r\left(t\right) \end{array} \end{array}$$

The generated membrane potential *u*(*t*) is then compared with a predefined threshold ϑ and output spike when *u*(*t*) > ϑ as is shown in Figure 1. In a multilayer feedforward spiking neural network architecture, instead of directly managing the non-differentiable spike neuron equations, SLAYER approximates the derivative of the spike function as a probability density function (PDF) of spike state changes. Further details of the model and its use in training the SNN can be found in Shrestha and Orchard (2018). With a good estimation PDF as the derivative term of spike change state, the SLAYER can easily derive the gradient of weights and delays in each layer from a feedforward SNN. This allows the network to adapt the developed gradient descent method for an optimization purpose such as ADAM (Kingma and Ba, 2015), RmsProp (Hinton et al., 2012).

### 2.3. Convolutional Recurrent Neural Network

The convolutional recurrent neural network (CRNN) structure has been well studied in the second generation of ANNs. The convolution operation in the ANNs usually acts as a spatial visual feature extractor that assumes features are in different levels of hierarchy. The recurrent structure introduces memory to the network and an ability to deal with sequential data dependently.

A significant design of the CRNN structure is the ConvLSTM structure (Shi et al., 2015) that was initially designed for forecasting precipitation. By replacing the general gate activation by the convolutional operation, the network is able to exploit an extracted 3D tensor as the cell state. The ConvLSTM was also evaluated on the moving MNIST (Srivastava et al., 2015) dataset and was shown to successfully separate the overlapping digits and predicted the overall motion with a high level of accuracy.

Another CRNN structure, CNN-LSTM, concatenates a CNN and an LSTM to formulate a collaborative network. The LSTM in the structure is placed behind a pretrained CNN that directly takes the output feature vector from the CNN as the input sequence. The implementation of this structure however is highly dependent on a well pre-trained CNN that was designed for the interest as the feature extractor. The CNN-LSTM has been proven to be powerful in many application domains such as acoustic scene classification (Bae et al., 2016), emotion recognition (Fan et al., 2016), action recognition (Wang et al., 2017) etc.

Over the past few years, researchers have successfully applied CRNN in medical applications (Wang L. et al., 2019), speech processing (Cakir et al., 2017; Tan and Wang, 2018), and music classification (Choi et al., 2017). Adopting a recurrent structure enables the neural network to encapsulate the global information while local features are extracted by the convolution layers. Yang et al. (2018) demonstrated a Convolutional LSTM network that was successfully evaluated on various action recognition datasets. The importance of using a CRNN structure in the application of human action recognition is that unlike action recognition in images, the same task in videos relies on motion dynamics in addition to visual appearance. Although CNNs and its variants like 3D convolution (Ji et al., 2013; Karpathy et al., 2014) achieves good performance, they still do not make sufficient use of temporal relations between frames. More recently, Majd and Safabakhsh (2019) designed a motion-ware ConvLSTM for the action recognition task which is an LSTM unit that considers the correlation of consecutive video frames in addition to the Spatio-temporal information.

However, in the SNN domain, the CRNN structure has not been widely investigated especially for the action recognition problem. One of the main challenges in developing a spiking CRNN is how to manage the training process of spiking neurons. Additionally, the consecutive information recurrency is difficult to achieve in the SNN since the traditional probabilistic based functions do not comply with spikes. In this paper, the SLAYER algorithm is used as an efficient, general supervised training mechanism for SNNs. Based on the spiking model of SLAYER, we design a network structure that can achieve both forward and recurrent information propagation.

## 3. Spiking Convolutional Recurrent Neural Network (SCRNN)

In this section, the novel system using SCRNN for action recognition is described. The fundamentals of 3D spiking convolution and the related SCRNN model are described in the following subsections.

### 3.1. Spiking Convolution Operation

**Figure 2:**
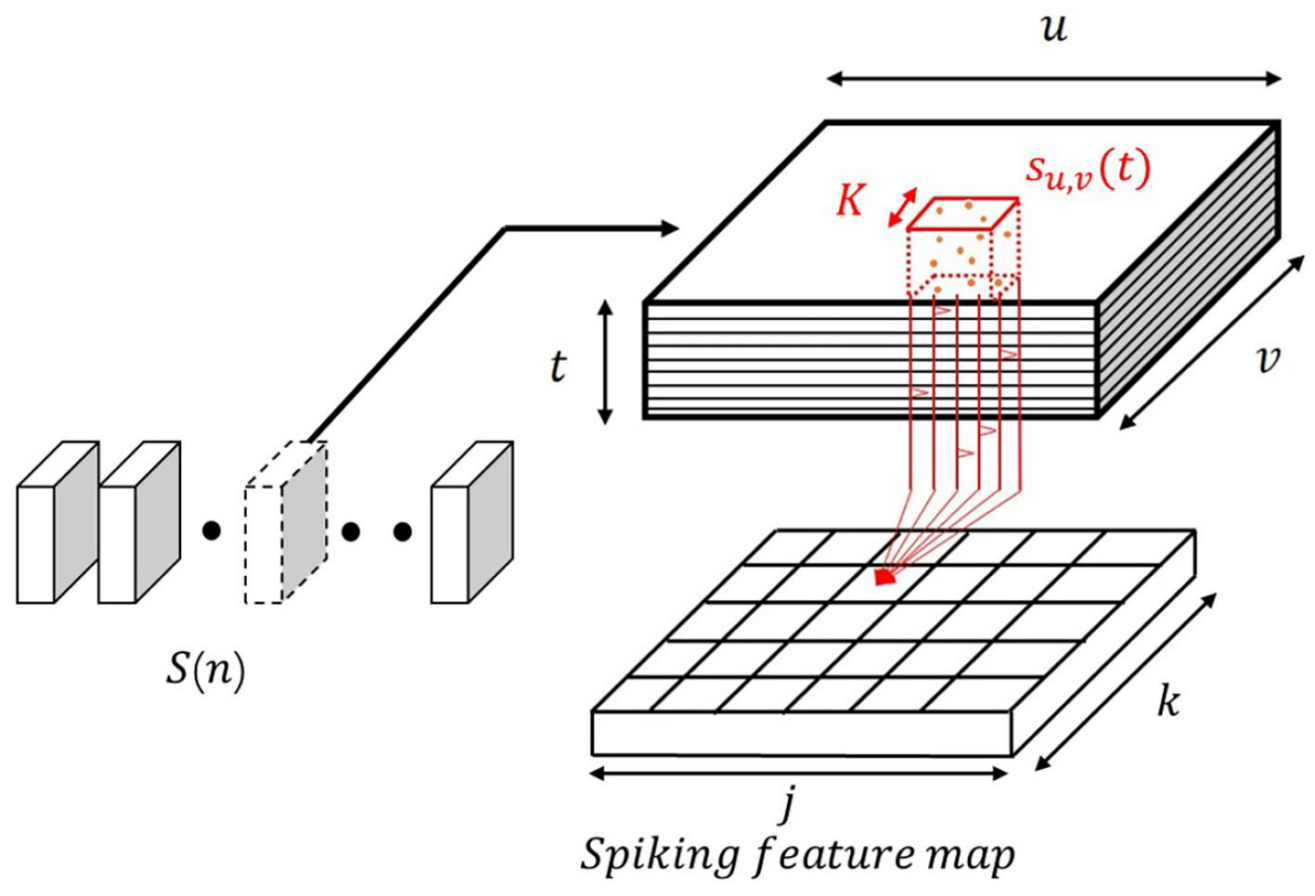
The 3D spiking convolution operation. Red: represents the spiking convolution through a defined 3D volume.

Consider an input sequence *S*(*n*), *n* = 0, 1, 2, ...*N* as is illustrated in Figure 2. At each time step, *S*(*n*) is a 3D tensor with shape {*u, v, t*} where *u* and *v* denote the width and height of each frame and *t* correspond to the pre-defined time resolution. For a given event-based video stream, it can be arbitrarily segmented into several tensors according to the desired temporal frequency. For example, for a 1.5 s 128 × 128 resolution events data stream with 30 ms temporal resolution and 1ms sampling time can form a input sequence *S*(*n*), *n* = 0, 1, 2, ...50. For each segments, the tensor shape is {128, 128, 30}.

The sampled input tensor *S*(*n*) with a shape of {*u, v, t*} is convolved with a 3D convolutional kernel to generate a spiking neuronal feature map. The spikes within an arbitrary kernel can be regarded as a bunch of spike trains *s*_*u, v*_(*t*) where each spike train corresponds to the spikes at a specific coordinate (*u, v*) within the temporal resolution window *t*. Each neuron in the feature map receives the spikes from the neurons in the 3D convolutional kernel. The spikes in the region of the kernel are integrated to generate membrane potential for a single neuron in the feature map. The neurons in a map detect the Spatio-temporal dynamic patterns in different 3D volumes. Unlike the standard feature map generated by CNN, the information at each coordinate in a spiking feature map is expressed by spike trains which can be considered as a spiking representation of detected patterns.

The convolutional kernel is highly overlapped to ensure the proper detection of features. The SRM neuron model is used to describe the 3D spiking convolution operation, which gathers all the input spikes from pre-synaptic neurons and outputs spike when the membrane potential reaches the pre-defined threshold. In the SLAYER, this is done by convolving the spike trains in the kernel with a spike response kernel followed by the threshold function. Each spike train will be transferred to the spike response signal then further to the membrane potential of the postsynaptic neuron. The process can be expressed as:

(2) $$\begin{array}{r} a_{u,v}\left(t\right)=s_{u,v}\left(t\right)*\sigma \left(t\right) \end{array}$$

(3) $$\begin{array}{r} u_{j,k}\left(t\right)=\overset{K}{\underset{m=1}{\sum }}\overset{K}{\underset{n=1}{\sum }}{\text{W}}_{m,n}a_{j+m-1,k+n-1}\left(t\right)+\left(s_{j,k}\left(t\right)*\nu \left(t\right)\right) \end{array}$$

(4) $$\begin{array}{r} s_{j,k}\left(t\right)=1\;\&\;u_{j,k}\left(t\right)=0\text{ when }u_{j,k}\left(t\right)\geq V_{thr} \end{array}$$

where **W** denotes to the synaptic weights, *u* and *v* are the vertical and horizontal coordinate index of the input tensor, *j* and *k* represents the vertical and horizontal coordinate in the feature map, and *K* represents the convolution kernel width and height.

The 3D spiking convolution can decompose the input event based data into several spatio-temporal pattern feature maps, where each spike in the map corresponds to a specific pattern. When multiple spiking convolution layers are used, the feature in a layer is a combination of several low level features extracted from the previous layer.

#### 3.1.1. SCRNN Cell

**Figure 3:**
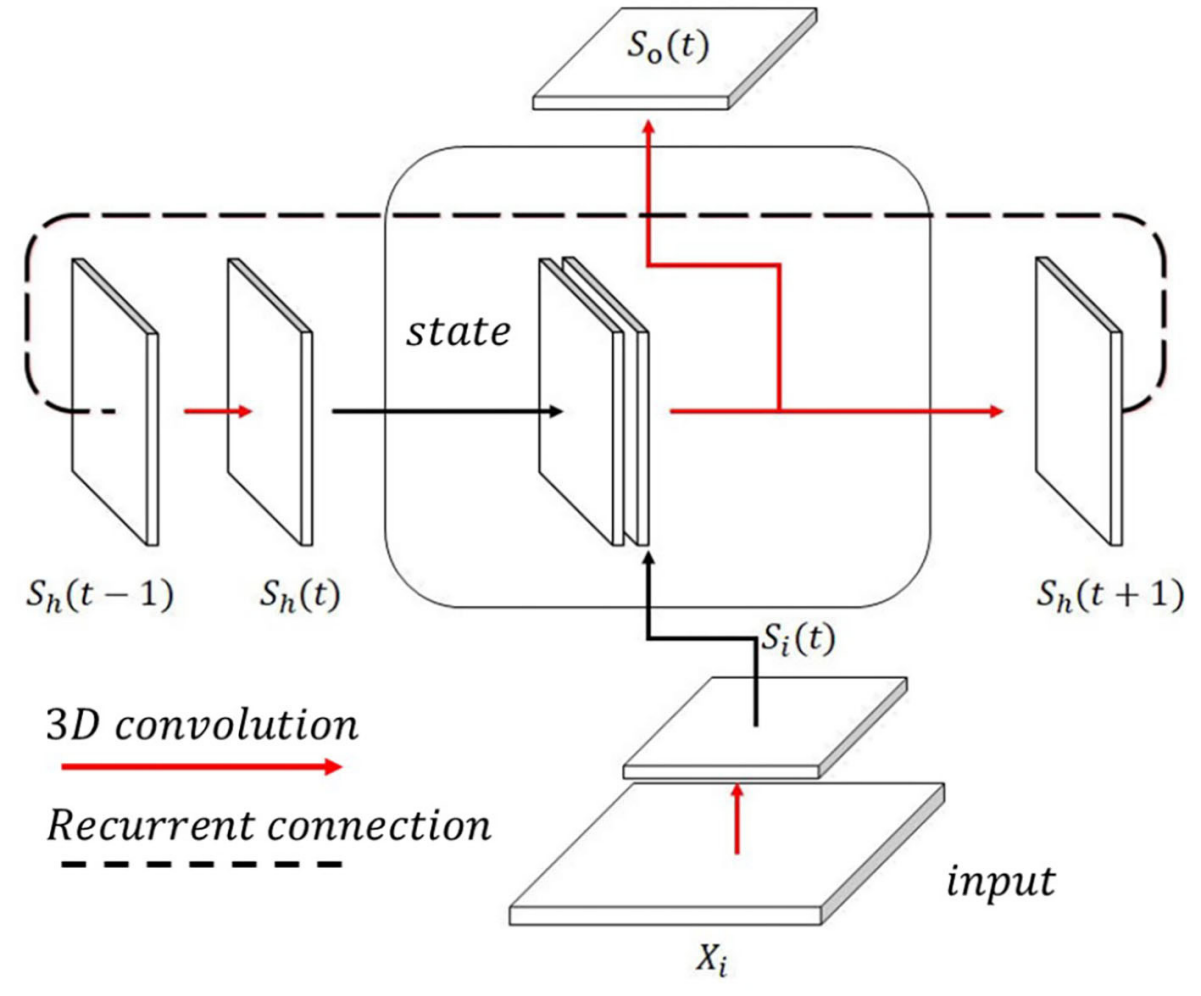
The proposed single SCRNN cell. The state spiking feature map and input feature map are combined in the cell with an output feature map recurrently connected to the cell.

The SCRNN cell is designed as the fundamental unit of the SCRNN system. The idea was inspired by the structure of the ConvLSTM cell (Shi et al., 2015). A graphical illustration of a single SCRNN cell is shown in Figure 3. The inputs to the cell comprise two parts. First is the spiking feature map generated by the outside events (e.g., a fragment from an event-based action data). The second part is the hidden spiking states which represent the fused feature map of previous states and the feature map generated by the current input. To ensure the state feature map has the same shape as the input, a padding technique is needed before the actual convolution operation, which means padding empty events (zeros) on the boundary of state maps. This can be viewed as the current state having no prior knowledge in terms of the region outside the current receptive field. At zero time index, the internal state needs to be initialized randomly or set empty which represents no prior knowledge at the beginning from the temporal perspective. Consequently, the 3D spiking convolution operation is applied to both input-to-internal state transitions and state-to-state transitions in an SCRNN cell. The future state to state transition is achieved by utilizing another 3D convolution layer that contains a pre-defined number of hidden neurons. Two feature maps are concatenated to form a single map. Then the spikes in the same kernel of the fusion map are accumulated and activated to generate the membrane potential signal for future states. Consider an input segment *X*_*i*_. The entire computation process within an SCRNN cell can be written as:

(5) $$\begin{array}{r} s_{i}\left(t\right)=\theta \left\{\sum W_{ih}\left(X_{i}*\sigma \left(t\right)\right)\right\} \end{array}$$

(6) $$\begin{array}{r} s_{h}\left(t\right)=\theta \left\{\sum W_{hi}\left(s_{h}\left(t-1\right)*\sigma \left(t\right)\right)\right\} \end{array}$$

(7) $$\begin{array}{r} s_{h}\left(t+1\right)=\theta \left\{\sum W_{hh}\left(s_{i}\left(t\right)*\sigma \left(t\right)+s_{h}\left(t\right)*\sigma \left(t\right)\right)\right\} \end{array}$$

(8) $$\begin{array}{r} s_{o}\left(t\right)=\theta \left\{\sum W_{ho}\left(s_{i}\left(t\right)*\sigma \left(t\right)+s_{h}\left(t\right)*\sigma \left(t\right)\right)\right\} \end{array}$$

where θ represents the thresholding operation. *W*_*ih*_, *W*_*hi*_, *W*_*hh*_, and *W*_*ho*_ denotes the weight input to state, state to input, state to state, and state to output, respectively. It can be seen from Equations (7) and (8) that the output of an SCRNN cell comprises two terms: *s*_*h*_(*t*+1) is the spiking states that can be used for future cells and the *s*_*o*_(*t*) represents the output spike train. The output from the cell represents the 3D feature map extracted from the current cell that allows the network to go deeper by using the *s*_*o*_(*t*) as the input of the next layer.

### 3.2. Spiking Convolutional Recurrent Neural Network

**Figure 4:**
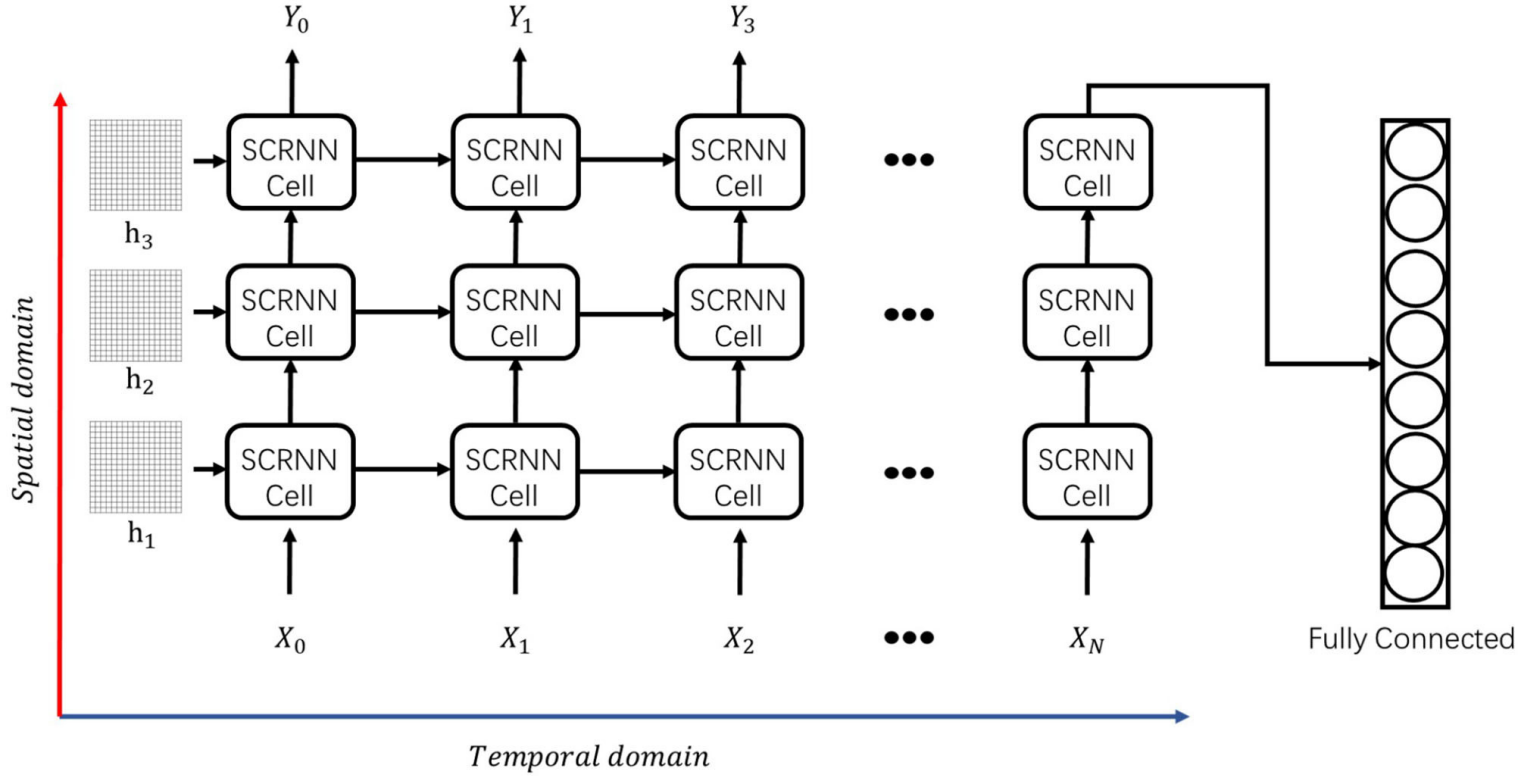
The proposed SCRNN structure which is comprised by prior defined individual SCRNN cells. The information going through the vertical direction in this figure is the spiking convolutional operation in the spatial domain. The information processing along with the horizontal direction in this figure is the recurrent process between the SCRNN cells which is in the temporal domain. *h*_1_, *h*_2_, and *h*_3_ is the initial feature map assumption prior to the zero index. *X*_*n*_ and *Y*_*n*_ represents the *n*_*th*_ input or the output sequences.

The overall SCRNN architecture shown in Figure 4 comprises a combination of single cells that are stacked in both the temporal and spatial processing domain. From a temporal point of view, the cells can process the input sequence separately using the internal state correlations. Furthermore, the input can be further decomposed by adding additional cells at each time step, thus allowing the network to form greater computational complexity and processing higher level spatial features. In other words, at a specific time step, the concatenated SCRNN cells (layers) can be treated as a standard spiking convolutional neural network wherein each input of an SCRNN cell is the output signal of the previous cell. It should be noted that additional initial states are needed for every added layer.

Similar to the conventional recurrent neural network, the SCRNN can also be unrolled to form a short-term feed-forward structure that increases the network parameter capacity. Unrolling a recurrent structure represents a trade-off between the network performance and the computational cost. Although theoretically the cells can be unrolled up to the length of the input sequence, the computational cost in the training process increases dramatically along with the number of cells. Moreover, to guarantee the network performance in terms of temporal information, the backpropagation through time (BPTT) (Werbos, 1990) is used, another factor that affects the training speed. BPTT calculates and accumulates errors across each time step, which can be computationally expensive as the number of time steps increase.

## 4. Experiment Results

**Figure 5:**
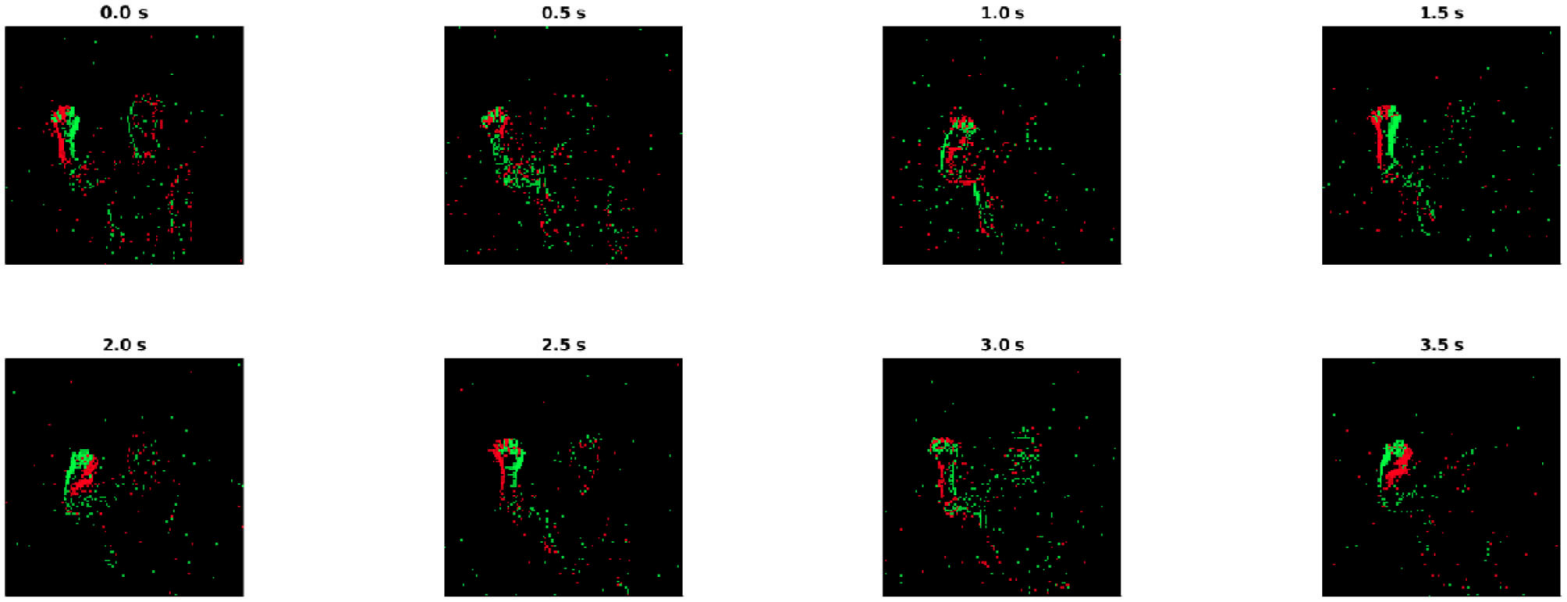
The demonstration of DVS gesture dataset with integral time of 0.5 s. The gesture showing in the example is hand waving. The green and red edges in each this figure represents the ON/OFF polarities of spikes.

In this section, the experimental result of action recognition using SCRNN will be presented. To validate the robustness of the SCRNN, we evaluated the network structure by performing the recognition task on the IBM DVS gesture dataset (Amir et al., 2017). The DVS gesture dataset comprises recordings of 29 different actors carrying out 10 different hand gesture actions. All recordings are captured by an Inilabs 128 × 128 dynamic vision sensor under three different lighting conditions. Each gesture sample has a duration of approximately 6 s. Figure 5 shows an example of hand waving gesture with 0.5 s integral time interval in nature light condition. The goal is to classify the gesture event video data into a corresponding label. The DVS gesture dataset is split as 1,176 samples for training and 288 samples for testing, as annotated. We construct a three layer SCRNN to solve this problem as is shown in Figure 4. The SRM response neuron parameters are shown in Table 1.

**Table 1:** The neuron parameter setting for the SCRNN simulation.

**ϑ_*neuron*_**	**τ_*neuron*_**	**τ_*ref*_**	***C*_*ref*_**	***tau*_*f*_**	***C*_*f*_**
5	10	1	2	1	1

The parameters define the standard neuron dynamics behavior which is used in all SCRNN networks. Where ϑ_*neuron*_ is the neuron firing threshold. τ_*neuron*_ is the neuron time constant, τ_*ref*_ is the neuron refractory time constant, *C*_*ref*_ is the refractory response scaling coefficient, *tau*_*f*_ is the neuron spike function derivative time constant, and the *C*_*f*_ is the neuron spike function derivative scaling coefficient.

As the gesture recognition is a many-to-one problem, only the output from the last layer and last time step SCRNN cell are considered for the loss calculation. The loss function used in this method is defined as the square error based on the number of spikes between the target and actual output in a time window according to Shrestha and Orchard (2018). Where the *S*_*o*_ denotes the output spike train of the last layer of SCRNN and Ŝ indicates the target spike train; the loss function *L* can be expressed as follows.

(9) $$\begin{array}{r} L=\frac{1}{2}\overset{N}{\underset{1}{\sum }}{\left(\int S_{o}\left(\tau \right)d\tau -\int Ŝ\left(\tau \right)d\tau \right)}^{2} \end{array}$$

where N is the number of output neurons of the last layer. At each time step, the error signal is calculated according to the current output spike count and target spike count. It should be noted that the backpropagation pipeline covers both spatial and temporal propagating routes through the recurrent connection. To save on computation resources, only 1.5 s out of 6 s of each gesture samples were used for the experiment. The input event sequence is integrated into several frames based on a pre-defined segmentation length *l*_*s*_. The segmentation length significantly affects the sparsity and the number of integrated frames. A small *l*_*s*_ results in a large number of sparse frames, on the contrary a chosen large *l*_*s*_ will reduce the number of frames but increase the number of events in each frame.

**Figure 6:**
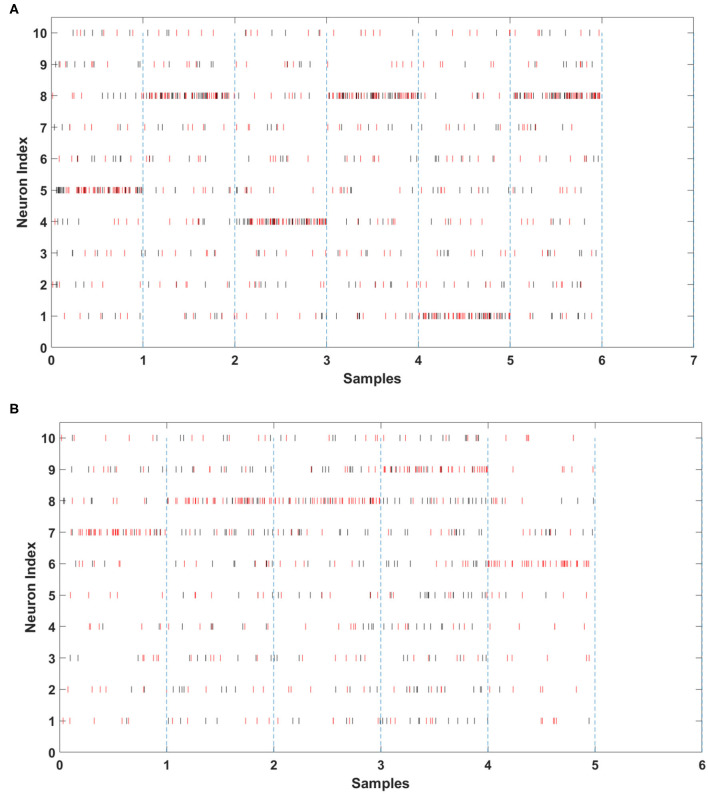
The last layer SCRNN output: **(A)** after training, **(B)** before training.

To evaluate the performance of SCRNN, we carried out different combinations of network parameters to perform the action recognition task. The following hyper-parameters were used in the experiments: Number of filters in the convolutional layer, the segmentation length (time resolution) *l*_*s*_, the target true spike count *Tg*_*True*_ and target false spike count *Tg*_*False*_. Figure 6 illustrates the output spike activities before and after the training of the last layer of the SCRNN. The vertical dash line in the figures simulates the time window of spiked that will be counted for an input sample. In other words, the spikes between two dash lines are the output from a single input instance. The output neuron index from 1 to 10 represents 10 different gesture classes. The red bars are target spike(labels) and the black bars are actual network output spikes. It should be noted that the loss for the SLAYER training algorithms is calculated from the error signal that was generated according to the difference between the number of actual output spikes from the network and the target spikes (*Tg*_*True*_ and *Tg*_*False*_). If the actual spike count of the output neuron match that of the target spike count then a correct prediction is implied. As shown in Figure 6A, the SCRNN has zero output before training and gradually learns to generate spikes that match the target spike in terms of the target spike quantity. Figure 6B demonstrates the output spike monitoring after-training the SCRNN. It can clearly be seen from Figure 6B that the actual spikes (shown in black) now have similar spike counts as target spikes (shown in red) for the input samples. It should be noted that the target spikes and actual spikes have different spike timings but similar spike counts in each window.

**Figure 7:**
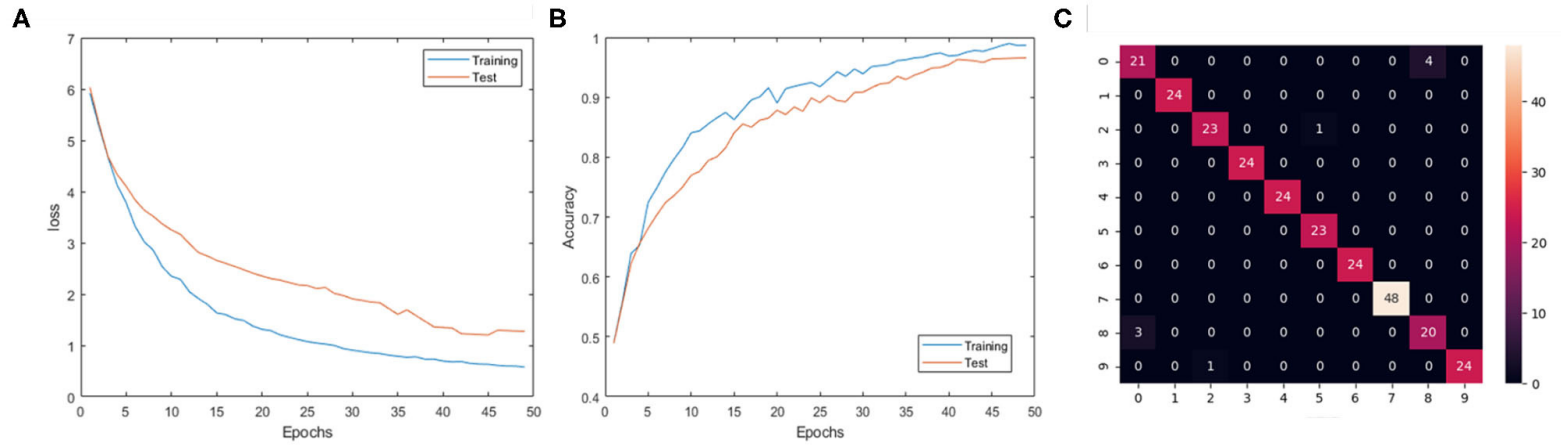
**(A)** The training and testing loss changes for 3 layer SCRNN with conv1: 5 × 5 × 32; conv2:3 × 3 × 64; conv3:3 × 3 × 128; *l*_*s*_ = 50 ms. **(B)** The training and testing accuracy changes for 3 layer SCRNN with conv1: 5 × 5 × 32; conv2:3 × 3 × 64; conv3:3 × 3 × 128; *l*_*s*_ = 50 ms. **(C)** The confusion matrix for 3 layer SCRNN with conv1: 5 × 5 × 32; conv2:3 × 3 × 64; conv3:3 × 3 × 128; *l*_*s*_ = 50 ms; The 0–9 represents the 10 categories of gestures. 0: hand clapping; 1:right hand wave; 2: left hand wave; 3: right arm clockwise; 4: right arm counter clockwise; 5: left arm clockwise; 6: left arm counter clockwise; 7: arm roll; 8: air drums; 9: air guitar.

The experiment results are shown in Table 2, where each listed architecture is simulated for 100 epoch over the full dataset. For each structure listed in the table, the accuracy is obtained by averaging the best testing accuracy among five repeated experiments with different random initialized weights. Among these experiments, the best testing accuracy of the 10 class gesture is 96.59%, with the 3 layer SCRNN structure with the first convolutional layer consisting of 32 5 × 5 convolutional filters, while the second and third convolution layer consist of 64 and 128 3 × 3 convolutional kernels, respectively. The *l*_*s*_ is 50 ms which represents a total of 1,000/50 = 20 time steps. The loss and training curve for the best network structure is shown in Figures 7A,B. This structure was also used to train the 11 class gesture (plus a random other gesture action) and obtained a testing accuracy of 90.28%.

**Table 2:** Comparisons of SCRNNs performance on DVS gesture dataset with different hyper-parameters.

***Conv*1**	***Conv*2**	***Conv*3**	***FC*1**	***FC*2**	***Tg*_*True*_**	***Tg*_*False*_**	***l*_*s*_(*ms*)**	**Trainacc (%)**	**Testacc (%)**
5 × 5 × 16	3 × 3 × 32	3 × 3 × 64	1,024	512	30	5	25	90.73	85.23
3 × 3 × 16	3 × 3 × 32	3 × 3 × 64	512	128	30	5	25	87.92	84.64
5 × 5 × 32	3 × 3 × 64	3 × 3 × 128	1,024	512	30	5	25	93.54	89.15
5 × 5 × 16	3 × 3 × 32	3 × 3 × 64	1,024	512	60	10	50	95.45	91.67
3 × 3 × 16	3 × 3 × 32	3 × 3 × 64	512	128	60	10	50	95.08	89.39
5 × 5 × 32	3 × 3 × 64	3 × 3 × 128	1,024	512	60	10	50	98.48	**96.59**
5 × 5 × 16	3 × 3 × 32	3 × 3 × 64	1,024	512	80	15	75	95.45	88.64
3 × 3 × 16	3 × 3 × 32	3 × 3 × 64	512	128	80	15	75	93.18	93.56
5 × 5 × 32	3 × 3 × 64	3 × 3 × 128	1,024	512	80	15	75	96.59	90.90

*Conv: Spiking convolutional layer; FC: Fully connected layer; Tg_True_: The preliminarily setting of target true spike count; Tg_Flase_: The preliminarily setting of target false spike count; l_s_(ms): Segmentation length; Trainacc: Training accuracy; Testacc: Testing accuracy*.

Thus, the loss can be very large at the start compared with a normal loss value since the network can have an empty output with untrained weights and delays. It was found that setting the *l*_*s*_ = 50*ms* produces the best result for the SCRNN structure which can be explained as follows. First, the time resolution is matched with the frame continuity for this dataset, which means the individual segmented frame can either contain limited or redundant information with *l*_*s*_ = 25*ms* or *l*_*s*_ = 75*ms*. This can possibly weaken the connection between the frames from the perspective of recurrent convolutional operation. Second, the spike emitting of neurons in each layer is important to the training process. A proper selection of *l*_*s*_ can ensure that the sparsity of frames guarantee the stability of the training process.

**Figure 8:**
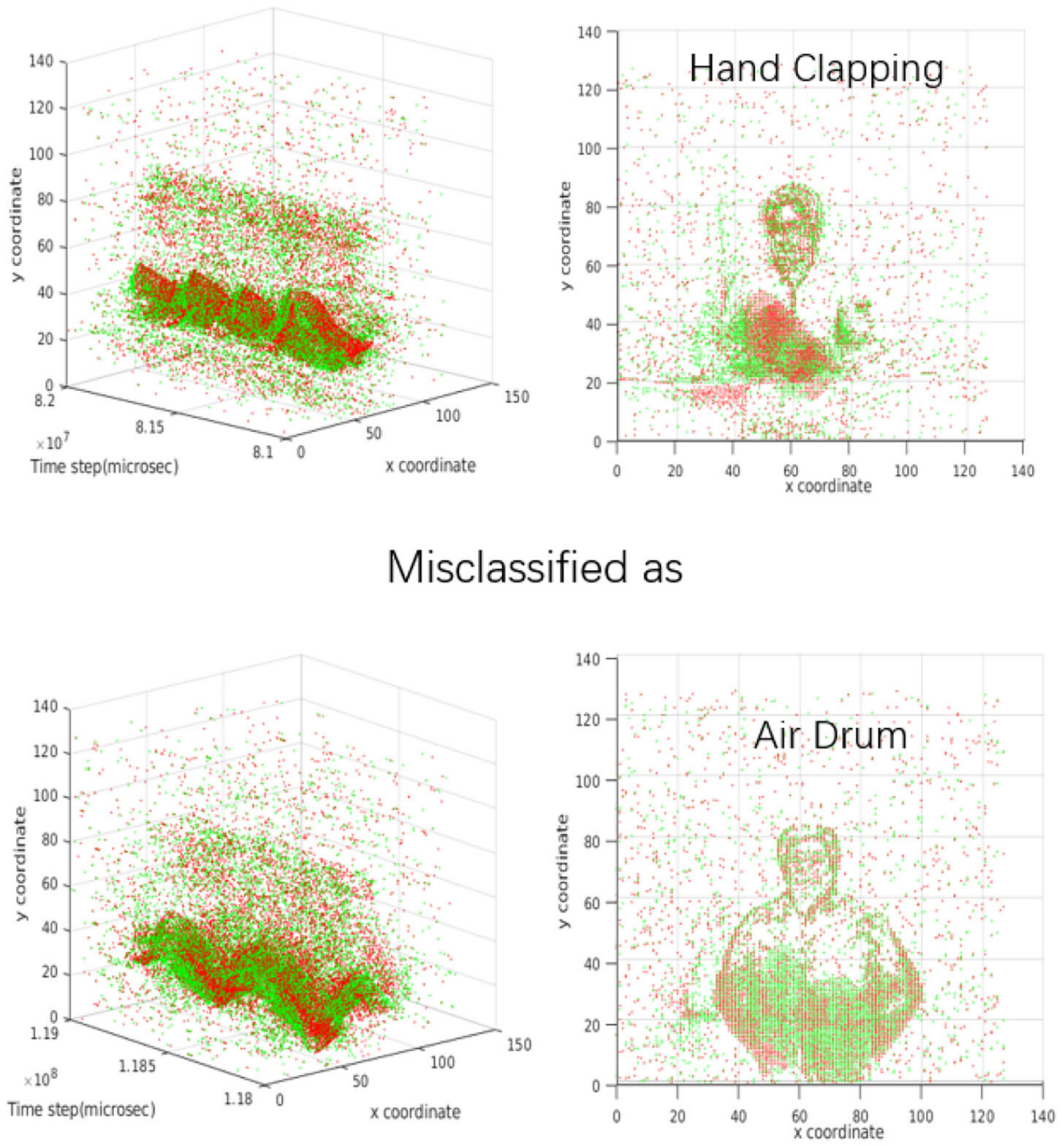
The example of 3 layer SCRNN misclassification case. The 4 figures demonstrate a similarity of event dynamics between the hand clapping gesture and air drum gesture. **Top left**: the 3D view of a hand clapping sample with duration of approximately 1 s. **Top right**: the 2D view of a hand clapping gesture that integrated all spikes within 1 s. **Bottom left**: the 3D view of a air drum gesture sample with duration of approximately 1 s. **Bottom right**: the 2D view of a air drum gesture that integrated all spikes within 1 s.

The confusion matrix in Figure 7C shows a detailed performance of the SCRNN for the 10 gesture recognition tasks. Note that the number of samples of arm roll is twice that of other gestures in the original dataset. It can be seen that the SCRNN achieved an overall good performance except for the confusion between the hand-clapping and air drums gesture where there are 3+4 = 7 total instances where SCRNN misclassified the hand clapping or air drum as the other. This is due to the dynamic similarity of these two gestures for some instances. Figure 8 demonstrates an example of misclassification which shows both the 3D and 2D view of the dynamics of these two gestures. From our observations, some hand-clapping and air drum gestures exhibit a strong similar spike change pattern which is a potential reason that leads to misclassification. This further matches our initial design purpose of SCRNN, which is an action dynamics sensitive event stream pattern based recognition network.

For comparison purposes, results from previously published work (Amir et al., 2017; Shrestha and Orchard, 2018; Wang Q. et al., 2019) on the IBM DVS gesture dataset is carried out which is shown in Table 3. It can be seen that the SCRNN approaches state-of-the-art recognition accuracy, surpassing the benchmark accuracy of IBM's work in 10 gesture classification task categories. The original work from IBM that ran on TrueNorth was trained with Eedn (Amir et al., 2017) and required extra filters and preprocessing before the CNN. On the other hand, the SCRNN takes the neuromorphic data directly from the sensor and the training process does not require any additional processing to the data. The SLAYER algorithms (Shrestha and Orchard, 2018) using CNN with a feedforward structure achieved an accuracy of 93.64% on average for the 11 class recognition. Although the SCRNN does not outperform the SLAYER based CNN network in 11 class classification, the SCRNN is still competitive at 90.28%. We conclude that this accuracy drop for the 11 class recognition task is due to the introduction of the additional class of the random gesture. The “other” class in the DVS gesture dataset consists of random samples and each of those are neither same as other samples nor do they fall into the first 10 categories. The SCRNN with designed recurrent convolution operation is found to be less effective in such types of training data. Although the SCRNN does not outperform the SLAYER based CNN network in 11 class classification, the SCRNN is still competitive at 92.01%. The pointnet++ (Wang Q. et al., 2019) processed individual event data using an MLP based feedforward neural network which achieved the best accuracy in both 10 and 11 category gesture recognition tasks. However, the pointnet++ is not a spiking based training algorithm, with less potential to be applied to neuromorphic hardware, and the DVS data in their method needs to be modeled as multiple points cloud with each spike {x,y,z} is fed into an MLP.

**Table 3:** Comparison of SCRNN gesture recognition results with previous work.

**Method**	**Type of processing**	**10 class**	**11 class**
IBM TrueNorth Eedn (Amir et al., 2017)	Spiking	96.49%	94.59%
SLAYER CNN (Shrestha and Orchard, 2018)	Spiking	Unknown	93.64%±0.49%
PointNet++ (Wang Q. et al., 2019)	Non-Spiking	97.08%	95.32%
SCRNN	Spiking	96.59%	92.01%

## 5. Effect of Recurrent Connection

**Figure 9:**
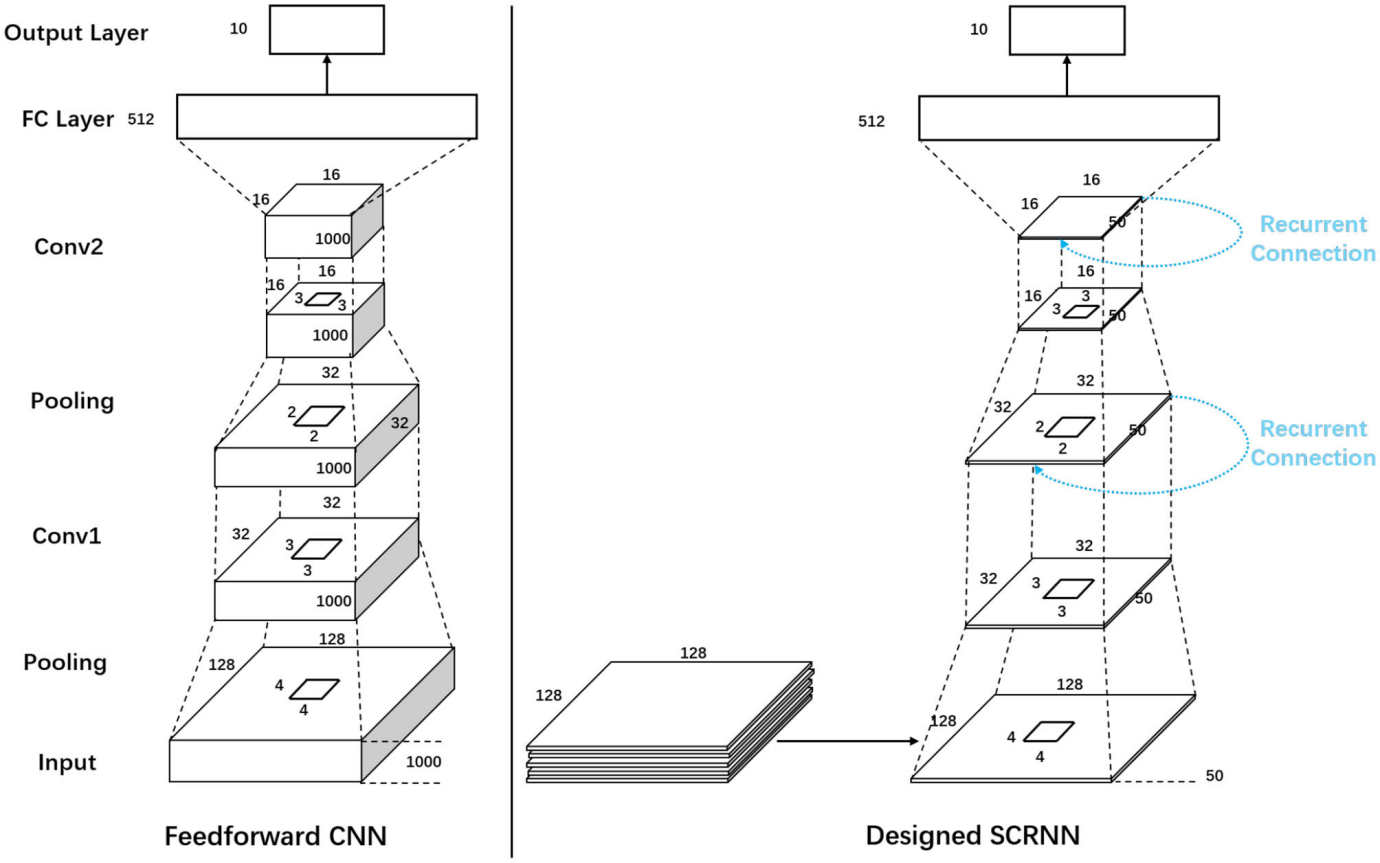
The network structure for the experiments of comparison between the feedforward Spiking Convolutional Neural Network and SCRNN.

To further demonstrate the effectiveness of SCRNN for the category-limited dynamic scene recognition. A mini-experiment is designed to directly compare the effect of the recurrence for the 10 class gesture recognition. A feedforward spiking convolutional neural network and an SCRNN is designed following a “same learning capacity rule” as is shown in Figure 9. The spike pooling operation was applied to reduce the computational cost. The pooling was done by reducing all the spikes in a pooling kernel into one over the spike presentation time. The two structures are exactly the same in neuron parameters, the number of neurons and number of layers except the SCRNN has a recurrent connection in each convolution layer. For both structures, with the segmentation length of *l*_*s*_, the first layer is a pooling layer with a kernel size of 4 × 4 × *l*_*s*_, which reduces the dimension of data from 128 × 128 × *l*_*s*_ to 32 × 32 × *l*_*s*_. The second layer is a convolutional layer that has a kernel size of 3 × 3 × *l*_*s*_ with 16 hidden neurons. The third layer is a pooling layer using 2 × 2 kernels to further reduce the dimension of each feature map to 16 × 16 × *l*_*s*_. The fourth layer is a convolutional layer with 32 hidden neurons with the kernel size of 3 × 3 × *l*_*s*_, which the output flattens and feeds into a fully connected layer with 5,256 neurons, followed by the output layer to perform the classification.

The feedforward CNN is different from the SCRNN in the training phase. For CNN, the first 1s event data of each sample with a temporal resolution of 1 ms (*l*_*s*_= 1,000) is used as the input data, which only needs to be fed to the network once per sample. The SCRNN takes the same length of input data in total for each sample but a segmentation length of *l*_*s*_ = 50 is selected to partition the input into 20 subsets. This represents that fact that the SCRNN needs to iteratively take the data to perform the recurrent processing.

**Figure 10:**
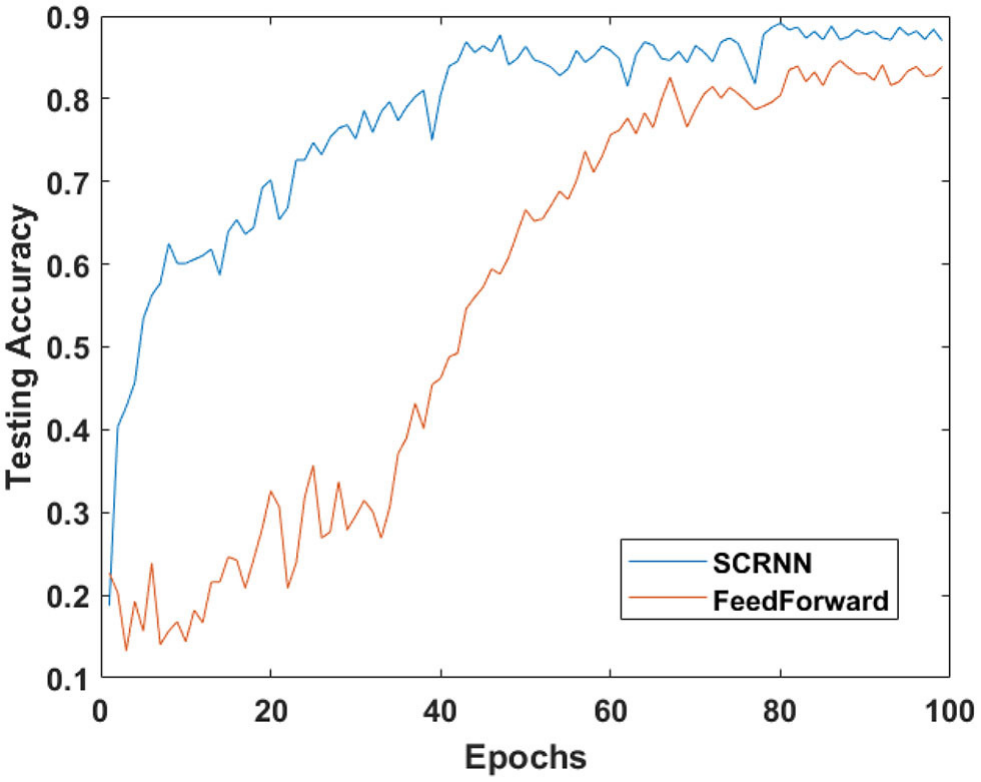
The testing accuracy curve for the designed experiments.

Both of the designed structures are trained for 100 epochs on five trials with different weight initializations, the averaged testing accuracy dynamics of these two experiments are plotted in Figure 10. The SCRNN compared to standard feedforward spiking CNN with a similar learning condition can provide a faster convergence speed. As shown in Figure 10, the averaged testing accuracy of SCRNN is stabilized after approximately 40 epochs while the CNN requires about 25 additional epochs to fully converge with the data. Furthermore, the SCRNN without the inference of the unknown class can provide a recognition accuracy of 88.64% on the 10 class gesture recognition in this particular structure, while the feedforward CNN only achieves 84.09%.

## 6. Conclusion

In this paper we presented a novel spiking convolutional recurrent neural network that was designed for efficient human hand gesture recognition. The individual cell is able to extract the spatial features by 3D spiking convolution operation and transferring the information recurrently.

The SCRNN is successfully deployed to the DVS 128 gesture dataset. The SCRNN tested on the IBM DVS gesture dataset achieved an averaged recognition accuracy of 96.59% for 10 category classification and 90.28% for 11 category classification. We have shown that the designed SCRNN compared to the standard feedforward CNN structure performs less competitively for the “unknown” class but has the advantage in terms of convergence speech and accuracy for the fixed number of categories.

We believe however, that the usage of SCRNN is not only limited to action recognition but can be extended to various dynamic scene recognition and prediction tasks. A further extension of this work could be a spiking-flownet-like network that is used for optical flow estimation (Dosovitskiy et al., 2015). Additionally, using new neuromorphic hardware with a low SWaP (Size, Weight and Power) profile, the SCRNN has the potential to be implemented as an efficient training algorithm for neuromorphic action recognition based applications. The SCRNN also has a strong potential to be implemented on the Loihi chip due to the use of the SLAYER algorithm.

## Data Availability Statement

Publicly available datasets were analyzed in this study. This data can be found here: http://www.research.ibm.com/dvsgesture/.

## Author Contributions

YX carried out the research and wrote the paper. JS and GD reviewed the paper. All authors contributed to the article and approved the submitted version.

## Conflict of Interest

The authors declare that the research was conducted in the absence of any commercial or financial relationships that could be construed as a potential conflict of interest.
